# Synthesis, Self-Assembly and In Vitro Cellular Uptake Kinetics of Nanosized Drug Carriers Based on Aggregates of Amphiphilic Oligomers of *N*-Vinyl-2-pyrrolidone

**DOI:** 10.3390/ma14205977

**Published:** 2021-10-11

**Authors:** Pavel P. Kulikov, Anna L. Luss, Levi C. Nelemans, Mikhail I. Shtilman, Yaroslav O. Mezhuev, Igor A. Kuznetsov, Oksana Yu. Sizova, Gunna Christiansen, Cristian P. Pennisi, Leonid Gurevich

**Affiliations:** 1 Department of Biomaterials, D. Mendeleev University of Chemical Technology of Russia, 125047 Moscow, Russia; p.kulikov.p@gmail.com (P.P.K.); shtilmanm@yandex.ru (M.I.S.); valsorja@mail.ru (Y.O.M.); Kuznetsov@muctr.ru (I.A.K.); oksana.sizova1991@mail.ru (O.Y.S.); 2 Centre for Strategic Planning of FMBA of Russia, 119121 Moscow, Russia; 3 Department of Materials and Production, Aalborg University, Skjernvej 4A, 9220 Aalborg, Denmark; levinelemans@gmail.com; 4 Department of Health Science and Technology, Aalborg University, Fredrik Bajers Vej 3B, 9220 Aalborg, Denmark; gunna@loke.dk (G.C.); cpennisi@hst.aau.dk (C.P.P.)

**Keywords:** drug delivery, nanocarriers, endocytosis

## Abstract

Development of nanocarrier-based drug delivery systems is a major breakthrough in pharmacology, promising targeted delivery and reduction in drug toxicity. On the cellular level, encapsulation of a drug substantially affects the endocytic processes due to nanocarrier–membrane interaction. In this study we synthesized and characterized nanocarriers assembled from amphiphilic oligomers of *N*-vinyl-2-pyrrolidone with a terminal thiooctadecyl group (PVP-OD). It was found that the dissolution free energy of PVP-OD depends linearly on the molecular mass of its hydrophilic part up to ${\bar{M}}_{n}$ = 2 × 10^4^, leading to an exponential dependence of critical aggregation concentration (CAC) on the molar mass. A model hydrophobic compound (DiI dye) was loaded into the nanocarriers and exhibited slow release into the aqueous phase on a scale of 18 h. Cellular uptake of the loaded nanocarriers and that of free DiI were compared in vitro using glioblastoma (U87) and fibroblast (CRL2429) cells. While the uptake of both DiI/PVP-OD nanocarriers and free DiI was inhibited by dynasore, indicating a dynamin-dependent endocytic pathway as a major mechanism, a decrease in the uptake rate of free DiI was observed in the presence of wortmannin. This suggests that while macropinocytosis plays a role in the uptake of low-molecular components, this pathway might be circumvented by incorporation of DiI into nanocarriers.

## 1. Introduction

The use of nanotechnology methods in drug delivery opens fundamentally new possibilities for regulating the concentration and exposure of an active component to pharmacological targets. Encapsulation of drugs into nanoscale aggregates is a modern approach which prolongs drug circulation, protects the drug from degradation and protects the organism from undesired toxic effects while achieving the required therapeutic effect [1,2,3]. Due to an increased chemical and colloidal stability in comparison with liposomes, nanosized aggregates based on polymers are becoming increasingly important for the development of drug delivery systems [4,5]. Usually, polymer aggregates are formed via self-assembly of chains, achieved by a combination of hydrophilic and hydrophobic blocks in a single macromolecule [6]. The most widespread drug carriers are based on polylactide [7,8,9], polyglycolide [10,11], and polyethylene oxide [12,13,14,15]. This fact is associated with their ease of preparation, low toxicity, and the ability to control the hydrophilicity (hydrophobicity) of the synthesized chains. Considerable prospects due to high biocompatibility and low toxicity [16,17] are associated with the use of carriers based on amphiphilic oligomers of *N*-vinyl-2-pyrrolidone [18,19,20,21]. Amphiphilic oligomers of *N*-vinyl-2-pyrrolidone can be produced by radical telomerization of *N*-vinyl-2-pyrrolidone in the presence of active chain transfer agents, among which thiols are of particular importance. n-Octadecyl mercaptan is commercially available and provides high colloidal stability of the resulting polymer nanosized aggregates. Apparently, n-octadecyl mercaptan is the most suitable among the studied long-chain thiols used for the simultaneous introduction of a hydrophobic fragment and control of the length of the hydrophilic block [22]. It should be noted that too short hydrophilic blocks negatively affect the solubility of PVP-OD, while too long hydrophilic blocks destabilize nano aggregates of PVP-OD. Furthermore, PVP of high molecular weight is excreted slowly and is toxic [16]. Therefore, the number average molecular weight ${\bar{M}}_{n}$ required for application of PVP-OD as drug carrier is in the range of 10^3^–10^4^, which corresponds to the number average degree of polymerization from about 9 to 90. 

The nanoaggregates of *N*-vinyl-2-pyrrolidone oligomers with a terminal thiooctadecyl group (PVP-OD) have been shown to form particles of 50 nm size capable of transporting curcumin into cell nuclei [23]. At the same time, the uptake mechanism and kinetics of the aggregates of PVP-OD ranging in size from 100 nm to 500 nm, which are usually formed because of self-assembly of chains without fractionation, remain unknown. In general, the kinetics and mechanism of uptake of nanoparticles by cells is often controversial and, possibly, specific for each type of carriers [24,25,26,27,28,29,30,31]. Although the clatrin-mediated endocytosis remains the most studied mechanism and considered to be the most common for drug delivery [24], macropinocytosis has recently attracted a substantial interest due its role in the development of oncological diseases [32] and in the emergence of drug resistance in the treatment of pancreatic cancer and breast cancer [33]. Inhibition of macropinocytosis has been shown to lead to the disappearance of drug resistance [33]. On the other hand, the ability of cell to uptake substantial quantity of the surrounding fluid via macropinocytosis (up to 2 µm in size) makes it interesting for drug delivery applications. This paper is focused on the in vitro uptake of synthetically available and non-toxic aggregates of amphiphilic oligomers of PVP-OD loaded with DiI dye used as a model hydrophobic drug by human glioblastoma (U87) and fibroblasts (CRL 2429).

## 2. Results and Discussion

### 2.1. Synthesis and Characterization of Nanoaggregates Based on PVP-OD

**Figure 1:**
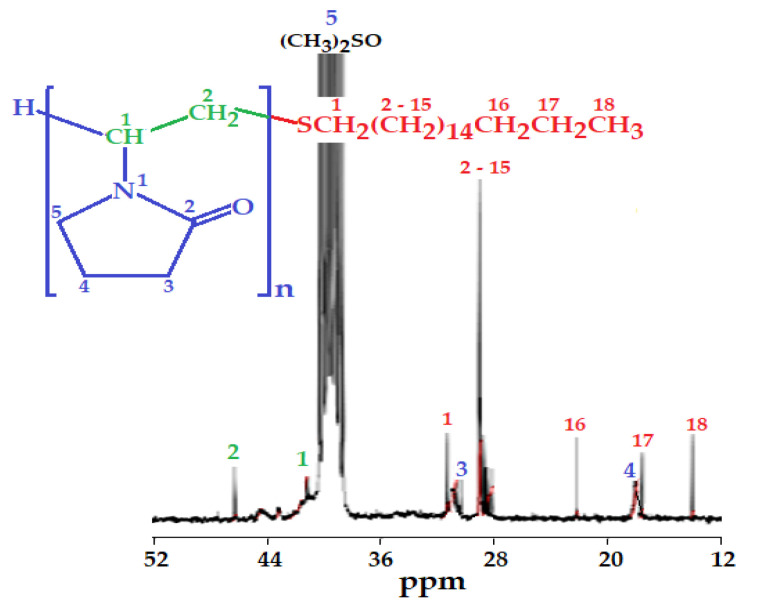
^13^C NMR spectra of the synthesized PVP-OD in DMSO-d6 showing the structure of the oligomers and peak assignment.

^13^C NMR studies of the synthesized PVP-OD showed no signals with a chemical shift exceeding 50 ppm, except for the 175 ppm singlet, corresponding to the carbon atoms of the amide carbonyl of *N*-vinyl-2-pyrrolidone residues in the oligomeric chain. Thus, the synthesized oligomers do not contain terminal nitrile groups, which indicates predominant binding of n-octadecylthio radicals formed at the initiation stage to *N*-vinyl-2-pyrrolidone [34]. As was recently shown [35], mercaptans and AIBN form a high-temperature redox system, which predetermines a high selectivity with respect to the introduced end groups. This is consistent with the peak assignment in the ^13^C NMR spectrum of PVP-OD, as shown in Figure 1.

**Figure 2:**
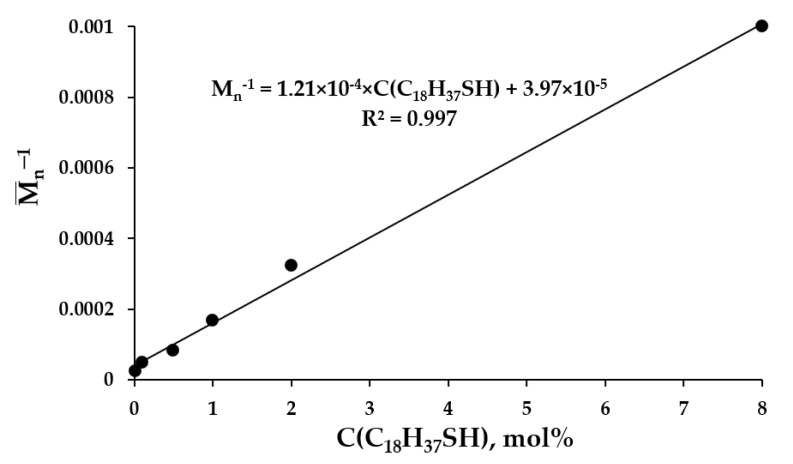
Dependence of the number average molecular weight PVP-OD on the concentration of n-octadecyl mercaptan in coordinates ${\bar{M}}_{n}^{-1}$ vs. $C\left(C_{18}H_{37}\mathrm{SH}\right)$.

The presence of both the hydrophobic n-octadecylthio moiety and the hydrophilic residues of *N*-vinyl-2-pyrrolidone in the PVP-OD chain creates prerequisites for self-organization of the oligomers in aqueous solution and formation of nanosized aggregates. With an increase in the number average molecular weight of the hydrophilic fragment (achieved by decreasing the concentration of n-octadecyl mercaptan in the reaction system) the solubility of PVP-OD increases as expected, which is manifested in an increase in the critical aggregation concentration (CAC). For synthesized PVP-ODs, a linear dependence of the inversed number average molecular weight ${\bar{M}}_{n}^{-1}$ as a function of n-octadecyl mercaptan concentration *C*(C_18_H_37_SH) was observed (Figure 2). This is a consequence of the Mayo Equation (1) [35,36], written for the case of a constant monomer concentration in the experimental series.

(1) $${\bar{M}}_{n}^{-1}={\bar{M}}_{n0}^{-1}+\xi C\left(C_{18}H_{37}\mathrm{SH}\right)$$

where ${\bar{M}}_{n}$ is the number average molecular weight of PVP-OD obtained in the presence of n-octadecyl mercaptan with a concentration of *C*(C_18_H_37_SH); ${\bar{M}}_{n0}$ is the number average molecular weight of PVP synthesized in the absence of n-octadecyl mercaptan; and ξ is a proportionality coefficient.

The values of ξ and ${\bar{M}}_{n0}$ calculated from the slope of the linear dependence shown in Figure 2 are 1.21 × 10^−4^ mol% ^−1^ and 2.52 × 10^4^, respectively.

**Figure 3:**
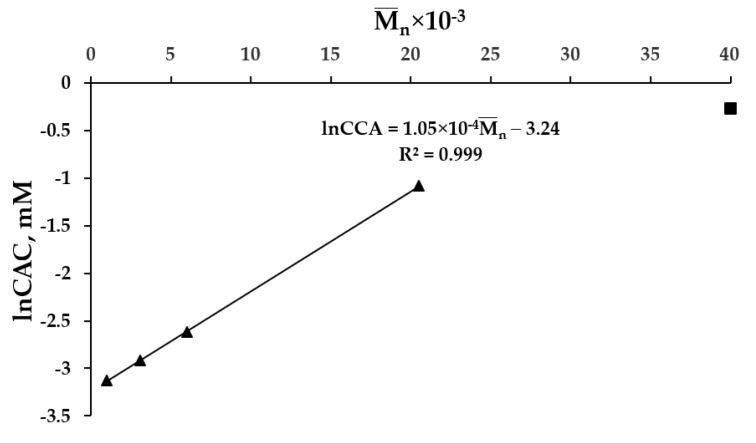
Concentration dependence of the critical aggregation concentration on the number average molecular mass plotted $\ln\left(\mathrm{CAC}\right)$ vs. ${\bar{M}}_{n}$.

Thus, by varying the concentration of n-octadecyl mercaptan in the reaction system, a series of PVP-ODs with different molecular weights of hydrophilic fragments and different CAC can be obtained. The dependence of the CAC on the number average molecular weight of the synthesized PVP-ODs is linear in the coordinates ln(CAC) vs. ${\bar{M}}_{n}$ up to ${\bar{M}}_{n}$ = 2 × 10^4^ (Figure 3). This shows that the tendency to form aggregates decreases with an increase in the length of the hydrophilic fragment of PVP-OD. It is manifested as an increase in CAC and broadening the concentration range where true aqueous solutions of PVP-OD exist. 

The observed linear dependence of ln(CAC) vs. ${\bar{M}}_{n}$ (Figure 3) can be explained in terms of the Van’t Hoff isotherm equation (2) [37], assuming a linear dependence of the free energy of solvation of PVP-OD vs. ${\bar{M}}_{n}$.

(2) $$\frac{{∆G}_{A}^{0}}{RT}=\ln\left(\mathrm{CAC}\right)\;=\frac{a}{RT}{\bar{M}}_{n}+\frac{b}{RT}$$

where: ${∆G}_{A}^{0}$ is standard free energy of aggregation of PVP-OD; CAC is the critical concentration of PVP-OD aggregation; *a*—free solvation energy per unit of the molecular weight for the hydrophilic part of PVP-OD; and *b* is the free energy of hydrophobic interactions of octadecylthio moieties which stabilize the aggregates.

As can be seen from Figure 3, *b* < 0, which predetermines the tendency of PVP-OD to self-assemble leading to the formation of aggregates; *a* > 0 indicates an increase in the solubility of PVP-OD in water with an increase in the length of the hydrophilic fragment and a corresponding increase in CAC.

Interestingly, the linear dependence of the free energy of solvation of PVP-OD on the molecular weight of the hydrophilic fragment can only be observed for small ${\bar{M}}_{n}$, when the contribution of interactions between *N*-vinyl-2-pyrrolidone residues to the free energy of dissolution remains rather constant. For large ${\bar{M}}_{n}$, on the other hand, when the density of hydrophilic units inside the polymer coil increases [38] and the contribution of polymer-solvent interactions drops, deviations from the linear dependence are observed. The latter presumable also leads to a break in the linearity of the ln(CAC) dependence ${\bar{M}}_{n}$ when the number average molecular weight exceeding 20 kDa. Therefore for PVP-OD, the transition from oligomers to polymers occurs in the ${\bar{M}}_{n}$ range from 20 to 40 kDa (Figure 3).

**Figure 4:**
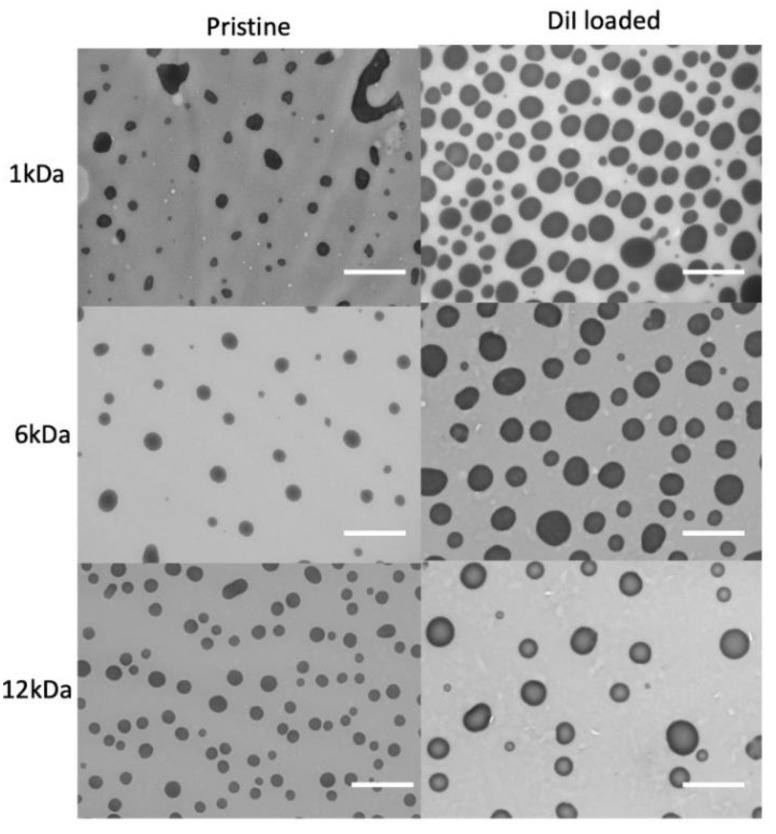
TEM micrographs of PVP-OD nanoaggregates formed by molecules with a different molecular weight of the hydrophilic part (1 kDa, 6 kDa, and 12 kDa) before and after DiI loading. Scale bars are 1 µm.

Figure 4 shows TEM micrographs of PVP-OD nanoaggregates obtained upon dissolution of PVP-OD molecules of different molecular weight in water and loading them with a hydrophobic DiI dye. The obtained aggregates appear to be dynamic structures, as indicated by round droplet-like shapes. While for the lowest molecular weight used (1 kDa) a certain stabilization of shape and size distribution was observed upon loading with DiI dye, generally, DiI loading just leads to an increase of the average size of aggregates. This indicates inclusion of DiI into hydrophobic regions of PVP-OD aggregates without their structural rearrangement. 

**Figure 5:**
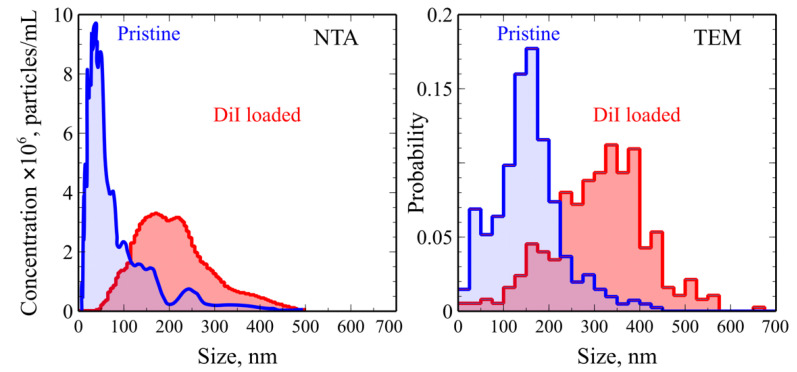
Size distribution of PVP-OD nanocarriers in DI water obtained from nanoparticle tracking analysis (NTA, left) and image analysis of TEM micrographs (TEM, right) for pristine aggregates of 6 kDa PVP-OD oligomers and PVP-OD aggregates loaded with DiI. The average number of particles per frame in NTA analysis was 44 and 41 for empty aggregates and the aggregates containing DiI, respectively. For image analysis, 407 pristine aggregates and 374 DiI-loaded aggregates were measured.

Nano-tracking analysis of PVP-OD aggregates with a number average molecular mass of ${\bar{M}}_{n}$= 6000 in water shows formation of aggregates with an average diameter of 105 nm and a mode of 37 nm was observed. Loading of DiI in the polymer aggregates led to an increase in their average diameter to 216 nm and the mode value to 172 nm, in line with the TEM observations (Figure 5), while maintaining the same average number of particles per frame in NTA measurements (44 and 41 for empty aggregates and the aggregates containing DiI, respectively). Thus, loading of DiI did not produce a strong effect on the number of nanoparticles in the system but rather led to an increase in their size. Interestingly, the size distribution changes upon DiI loading observed with NTA and TEM are very similar; however, there is a shift towards larger sizes in TEM. This can be explained by flattening of aggregates upon drying on the grid as well as the effect of the contrast media. A similar trend was observed for particles formed of PVP-OD with 1 kDa, 3 kDa and 12 kDa molecular mass. Therefore, it can be assumed that DiI is incorporated into the hydrophobic regions of aggregates formed by the octadecyl sections of amphiphilic PVP-OD, hence changing just the size of the aggregates and not their morphology. It should be noted that although number of works have addressed drug loading into nanosized aggregates based on amphiphilic PVP [22,23], the conservation of the overall morphology and the number of particles accompanied by an increase in their average size upon drug loading has not been shown earlier. 

**Figure 6:**
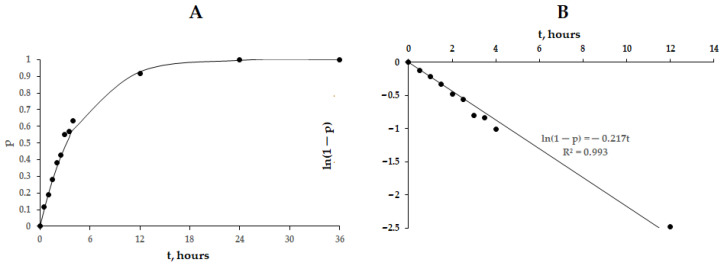
Release kinetics of DiI from nanosized PVP-OD aggregates: (**A**) time dependence of the DiI concentration in aqueous phase normalized to the maximum concentration. (**B**) Same data linearized by plotting $\ln\left(1-p\right)$ vs. *t*. Solid lines in both panels show a fit of the experimental data to a first order kinetic equation with a rate of 0.217 h^−1^.

Drug release experiments showed that DiI-loaded nanosized PVP-OD aggregates exhibit slow release of DiI in water. Compared to dissolution of pure DiI, where the maximum concentration was reached within 30 min, DiI release from nanoaggregates of 6 kDa PVP-OD took 18 h to reach the maximum concentration (Figure 6A).

It can be seen that DiI release from the nanoaggregates follows a first order kinetic equation:

(3) $$\frac{dA}{dt}=k\left(A_{max}-A\right)$$

where $A\;and\;A_{max}$ are the optical absorption and maximum optical absorption at saturation measured at 500 nm wavelength, *k* is the release rate and *t* is time. 

Integration of Equation (3) leads to Equation (4) which yields a linear graph when plotted as $\ln\left(1-p\right)$ vs. *t*:

(4) $$\ln\left(1-\frac{A}{A_{max}}\right)=-kt$$

where $p=\frac{A}{A_{max}}$ shows the progression of DiI release from the PVP-DO aggregates. 

The release rate calculated by fitting Equation (4) to the experimental data plotted as $\ln\left(1-p\right)$ vs. *t* yielded a value of 0.217 h^−1^ (0.0036 min^−1^), see Figure 6B. 

Thus, PVP-OD nanoaggregates can be used to form a drug delivery system capable of slow release of hydrophobic drugs. 

### 2.2. Cellular Uptake of PVP-OD Nanoaggregates

**Figure 7:**
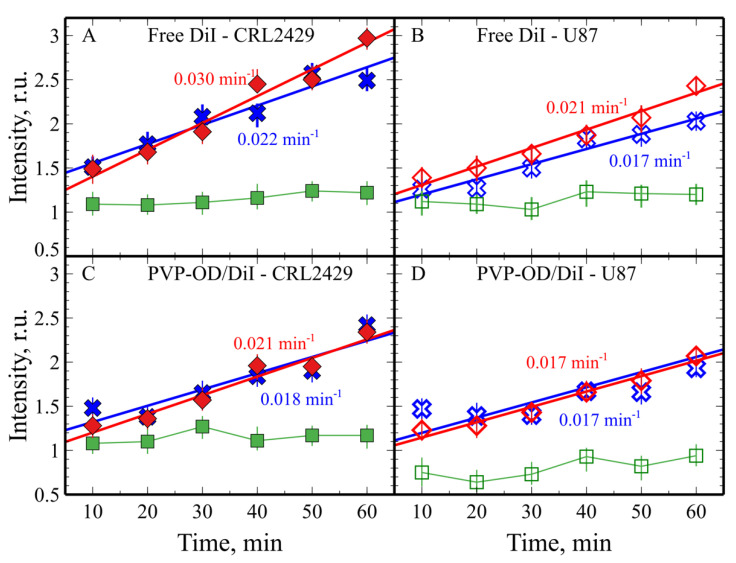
Uptake kinetics of free DiI (**A**,**B**) and DiI-loaded PVP-OD nanoparticles (**C**,**D**) for CRL 2429 fibroblasts (**A**,**C**) and U87 glioblastoma (**B**,**D**) cell lines and in the absence of endocytosis inhibitors (red), in the presence of wortmannin (blue) and dynasore (green) measured as the dependence of relative DiI fluorescence on the incubation time. Trend lines and the respective rate coefficients for the uptake without inhibitors, in the presence of wortmannin and in the presence of dynasore are shown in red, blue, and green, respectively.

Figure 7 shows uptake curves for free DiI and DiI loaded into nanosized PVP-OD aggregates. The experiments were carried out using cell cultures of fibroblasts and glioblastoma cells with and without endocytose inhibitors. It should also be noted that within the studied time range of 60 min, the kinetic curves can be satisfactory approximated with linear dependences. 

In all the cases, the administration of the inhibitor of dynamine-dependent endocytosis, dynasore, led to an abrupt decrease in the fluorescence intensity, indicating a compromised uptake of DiI. The slight increase in fluorescence with time in the presence of dynasore (Figure 7, green curves), observed in all cases, can be explained by the adsorption of DiI on the cellular membrane followed by slow passive transport into the cell (due to diffusion across the cell membrane). 

The uptake in the absence of endocytosis inhibitors showed approximately linear behavior in the time range studied with the rates of 3.0 × 10^−2^ min^−1^ and 2.1 × 10^−2^ min^−1^ for pure DiI by CRL2429 and U87 cells, respectively (Figure 7A,B, red experimental points and trend lines). In the presence of wortmannin [39], the endocytosis rate decreased down to 2.2 × 10^–2^ min^–1^ and 1.7 × 10^−2^ min^–1^ for CRL2429 and U87 cells, respectively (Figure 7A,B, blue experimental points and trend lines). Thus, for pure DiI, macropinocytosis (or possibly, receptor-mediated endocytosis) makes a certain contribution to DiI uptake. 

The observed uptake kinetics of DiI-loaded PVP-OD nanoaggregates showed slower uptake rates as compared to free DiI, on average 2.1 × 10^−2^ min^−1^ and 1.7 × 10^−2^ min^−1^ for CRL2429 and U87 cells (Figure 7C,D, red experimental points and trend lines). In contrast to the uptake of pure DiI, the addition of wortmannin had a very minor effect on the uptake rate of DiI loaded nanosized aggregates (Figure 7C,D, blue points and trend lines). As can be seen, the rate constants of the uptake of pure DiI in the presence of wortmannin are practically identical to the values for the uptake of DiI included in nanosized aggregates. Thus, loading of DiI in nanosized PVP-OD aggregates had the same effect on the rate of dye uptake as the presence of an inhibitor of receptor-mediated endocytosis and macropinocytosis, wortmannin. It can be therefore concluded that loading of DiI into nanosized aggregates of PVP-OD leads to blocking of macropinocytosis. Although the exact mechanism of this phenomenon is beyond the scope of this paper, it can be argued that free DiI can easily penetrate the cell membrane and spread within its hydrophobic region. Since macropinocytosis involves ingestion of large patches of cell membrane and their transport into the cell interior, free DiI can be also transported and distributed throughout the cell using this mechanism. DiI loaded into nanocarriers cannot exchange with the membrane so easily and therefore is mainly taken up together with the nanocarriers through dynamic-dependent endocytosis. This phenomenon can be of interest and importance when developing a drug delivery system based on nanocarriers. While loading drugs into nanosized PVP-OD aggregates brings a number of advantages associated with an increased circulation time and reduced toxicity [17,40], certain uptake mechanisms might be suppressed. It should be noted that both inhibitor-free and wortmannin-inhibited cellular uptake exhibit rates 5–8-fold higher than the observed release rates of DiI from PVP-OD nanocarriers (Figure 7). This confirms the active uptake due to endocytosis as the main uptake mechanism. On the other hand, the uptake rates observed in the presence of the dynamin inhibitor dynasore are similar to the observed release rates as DiI diffusion into cell membranes and its release from nanocarriers become the limiting factors. 

**Figure 8:**
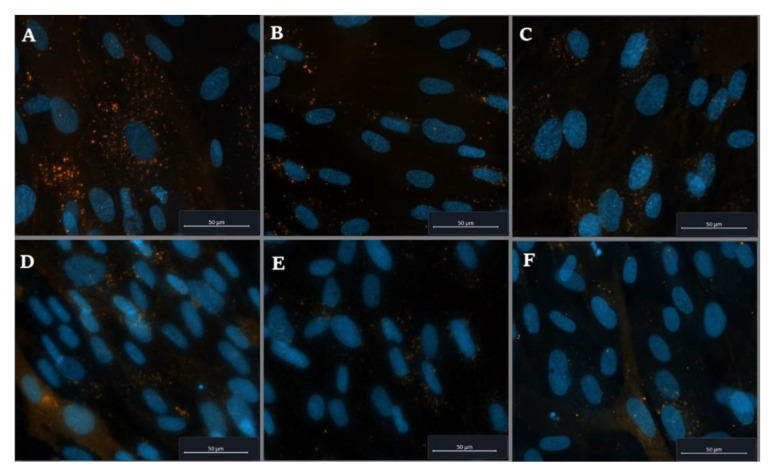
Composite fluorescence micrographs of CRL2429 fibroblasts after uptake of pure DiI (**A**—without inhibitors; **B**—in the presence of dynasore; **C**—in the presence of wortmannin) and DiI loaded nanocarriers (**D**—without inhibitors; **E**—in the presence of dynasore; **F**—in the presence of wortmannin). Cell nuclei are shown in blue as nucleic dye Hoechst 33258 was used. Orange color corresponds to the fluorescence at the DiI emission wavelength.

**Figure 9:**
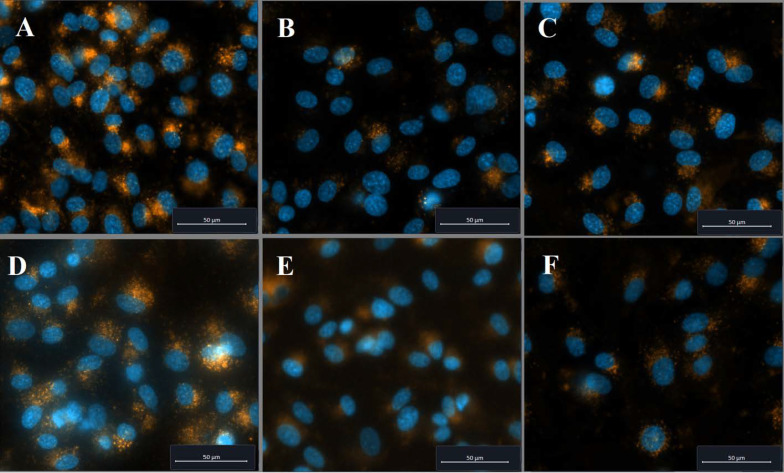
Composite fluorescence micrographs of the U87 cell line after uptake of pure DiI (**G**—without inhibitors; **H**—in the presence of dynasore; **I**—in the presence of wortmannin) and DiI loaded nanocarriers (**J**—without inhibitors; **K**—in the presence of dynasore; **L**—in the presence of wortmannin). Cell nuclei are shown in blue as nucleic dye Hoechst 33258 was used. Orange color corresponds to the fluorescence at the DiI emission wavelength.

Fluorescence microscopy data for both free DiI and DiI-loaded PVP-OD nanosized aggregates in both cell cultures confirmed accumulation of the fluorophore in the lysosomes around nuclei (Figure 8 and Figure 9). It is also seen that the concentration of endosomes significantly drops with the introduction of dynasore, indicating dynamine-dependent endocytosis as the main uptake mechanism.

Thus, the use of aggregates of amphiphilic *N*-vinyl-2-pyrrolidone oligomers as nanoscale carriers makes it possible to minimize the contribution of macropinocytosis and receptor-mediated endocytosis in general, due to the size and inert surface of the aggregates. Although this effect was shown using a model hydrophobic drug—DiI dye, it can be assumed that a similar effect can be observed for other pharmacologically active compounds. This can be particularly important for transiting from systemic application of free drugs, mainly used at the present time, to novel drug delivery systems with encapsulated drugs, in particular, as macropinocytosis is not only involved in supplying the nutrition for cancer cells but is also instrumental in formation of drug resistance of cancer cells [33,41,42]. 

## 3. Materials and Methods

**Scheme 1:**
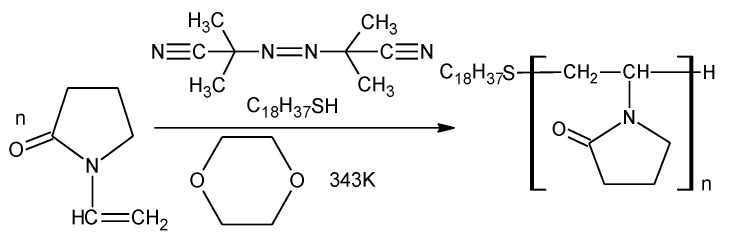
Synthesis of amphiphilic telomer *N*-vinyl-2-pyrrolidone with a terminal thiooctadecyl group.

PVP-OD of different molecular weights was synthesized using a radical polymerization reaction of *N*-vinyl-2-pyrrolidone in a 1,4-dioxane solution at 343 K, initiated by azobisisobutyronitrile (AIBN) in the presence of octadecyl mercaptan (Scheme 1). *N*-vinyl-2-pyrrolidone and 1,4-dioxane were purchased from Sigma-Aldrich (St. Louis, MO, USA).

For the synthesized PVP-OD, the number-average molecular weights and the critical aggregation concentration (CAC) were determined using the methods of end-group analysis and fluorescence spectroscopy (Hitachi 650-10S, Tokyo, Japan)), using earlier described methodologies [22]. The formation of nanosized aggregates was carried out using PVP-OD with a number average molecular weight of 6 kDa. The structure of PVP-OD was characterized by ^13^C NMR spectroscopy (AMX 400, Bruker, Karlsruhe, Germany) in DMSO-d6 (with 72 h signal accumulation). 

Pristine nanoaggregates were prepared by dissolving PVP-OD oligomer in Milli-Q water. DiI fluorescent dye (Life Technologies) was used as a model hydrophobic drug. To load the nanoaggregates with DiI, the dye was first dissolved in chloroform to a concentration of 10 mg/ml and added dropwise to 100 mg/mL aqueous colloidal solution of PVP-OD under ultrasonication (the test tube was placed on ice to avoid overheating). Then chloroform was removed by vacuum distillation, the system was centrifuged at 5000 rpm, followed by freezing and lyophilization of the supernatant (Alpha 1-4 LD plus, Martin Christ, Germany). The size distribution of the obtained nanoaggregates was studied with nanoparticle tracking analysis using a Nanosight LM10 (Malvern Pananalytical) equipped with a 642 nm laser source. For morphology studies, the samples were deposited on a carbon-coated glow discharged 400 mesh nickel grids, stained with one drop of 0.5% phosphotungstic acid pH 7.0, and imaged with Jeol JEM-1010 transmission electron microscope operating at 60 kV. 

For DiI release study, 0.1 ng of 6 kDa PVP-OD nanoaggregates loaded with DiI was suspended in 10 ml of distilled water and dialyzed against distilled water at 37 ℃ using a membrane with a molecular weight cut-off of 12 kDa. The absorption of the dialysis fluid was measured at 555 nm using UV Vis spectrometer UNICO 2804 (Princeton, NJ, USA).

Human primary foreskin fibroblasts (CRL 2429) and the human glioblastoma cell line (U87) were obtained from American Type Culture Collection (ATCC). Cells were grown in Dulbecco’s modified Eagle Medium (DMEM F12, Thermo Scientific Fisher) supplemented with 100 U/mL penicillin, 10 mg/mL streptomycin, and 10% FBS in a humid environment, 5% CO_2_ at 37 °C [23]. The cells were then seeded in 96 well plates so that the cell density was about 5 × 10^3^ cells × cm^−2^. For each type of cells, the uptake kinetics of DiI was measured both for DiI solution and DiI loaded into the nanocarriers in the absence of endocytosis inhibitors, as well as in the presence of 0.1 µM wortmannin (Sigma-Aldrich), to inhibit macropinocytosis [43], or 80 µM dynasore (Sigma-Aldrich), to inhibit clathrin-mediated endocytosis [44]. Cellular tests were performed in accordance with the previously described method [23], replacing curcumin with DiI. Briefly, the cell culture medium was replaced with DMEM F12 medium with or without inhibitors and incubated for 30 min, followed by washing with phosphate-buffered saline (PBS). Afterwards, the DMEM F12 medium supplemented with either free DiI or DiI-loaded nanocarriers was added to the wells for a prescribed time followed by washing with PBS and counterstained with Höchst 33258 nucleic dye (Thermo Fisher Scientific). The plates were then transferred to a Wallac Victor II microplate reader (Perkin Elmer) where the fluorescence intensity was separately recorded at the DiI and Höchst 33258 emission wavelength with top illumination. The relative fluorescence intensity of DiI was obtained by normalization to that of Höchst 33258 dye. Micrographs of cell cultures at the different incubation times were obtained using an Axio Observer.Z1 inverted microscope (Carl Zeiss, Jena, Germany).

## 4. Conclusions

We demonstrated that the use of n-octadecyl mercaptan as a chain transfer agent makes it possible to synthesize amphiphilic polymers via radical polymerization of *N*-vinyl-2-pyrrolidone in a 1,4-dioxane solution. The chain transfer to n-octadecyl mercaptan obeys the Mayo equation and provides the ability to control the molecular weight of the hydrophilic fragment of amphiphilic PVP-OD molecules. For oligomeric PVP-OD, an linear relationship between the ln(CAC) and the number average molecular mass was observed indicating a linear change in the free energy of solvation with an increase in the length of the hydrophilic fragment. The synthesized PVP-OD at a concentration above CAC forms stable nanosized aggregates capable of incorporating the DiI dye for all molecular weights studied. The aggregates maintained round shape and were found just to increase their average size upon DiI loading. The DiI-loaded PVP-OD nanoaggregates were observed to release DiI slowly into aqueous phase on a scale of 18 h, which indicated that they are suitable for delivery of hydrophobics drugs. 

In vitro cellular uptake of both free DiI dye and DiI-loaded PVP-OD aggregates in primary fibroblasts (CRL 2429) and in glioblastoma cells (U87) exhibited a linear time dependence during the first 60 minutes. Dynasore was found to effectively inhibit the penetration of both pure DiI and DiI-loaded nanosized aggregates, which indicates the dynamin-dependent endocytosis in both cases. Wortmannin, which inhibits macropinocytosis, does not change the uptake rate of DiI-loaded nanocarriers in the cell, but decreases the rate of uptake of the pure dye. Exclusion of certain endocytic pathways for drugs loaded into nanocarriers can be a general phenomenon which should be considered when developing novel nanocarrier-based drug delivery systems.

## Data Availability

The data presented in this study are available on request from the corresponding author.
